# MicroRNA-99 Family Members Suppress Homeobox A1 Expression in Epithelial Cells

**DOI:** 10.1371/journal.pone.0080625

**Published:** 2013-12-03

**Authors:** Dan Chen, Zujian Chen, Yi Jin, Dragan Dragas, Leitao Zhang, Barima S. Adjei, Anxun Wang, Yang Dai, Xiaofeng Zhou

**Affiliations:** 1 Center for Molecular Biology of Oral Diseases, College of Dentistry, University of Illinois at Chicago, Chicago, Illinois, United States of America; 2 Department of Oral and Maxillofacial Surgery, the First Affiliated Hospital, Sun Yat-Sen University, Guangzhou, China; 3 Department of Anatomy and Cell Biology, Rush University Medical Center, Chicago, Illinois, United States of America; 4 Department of Oral and Maxillofacial Surgery, Nan Fang Hospital, Southern Medical University, Guangzhou, China; 5 Department of Bioengineering, College of Engineering, University of Illinois at Chicago, Chicago, Illinois, United States of America; 6 UIC Cancer Center, University of Illinois at Chicago, Chicago, Illinois, United States of America; 7 Department of Periodontics, College of Dentistry, University of Illinois at Chicago, Chicago, Illinois, United States of America; CNRS UMR7275, France

## Abstract

The miR-99 family is one of the evolutionarily most ancient microRNA families, and it plays a critical role in developmental timing and the maintenance of tissue identity. Recent studies, including reports from our group, suggested that the miR-99 family regulates various physiological processes in adult tissues, such as dermal wound healing, and a number of disease processes, including cancer. By combining 5 independent genome-wide expression profiling experiments, we identified a panel of 266 unique transcripts that were down-regulated in epithelial cells transfected with miR-99 family members. A comprehensive bioinformatics analysis using 12 different sequence-based microRNA target prediction algorithms revealed that 81 out of these 266 down-regulated transcripts are potential direct targets for the miR-99 family. Confirmation experiments and functional analyses were performed to further assess 6 selected miR-99 target genes, including mammalian Target of rapamycin (mTOR), Homeobox A1 (HOXA1), CTD small phosphatase-like (CTDSPL), N-myristoyltransferase 1 (NMT1), Transmembrane protein 30A (TMEM30A), and SWI/SNF-related matrix-associated actin-dependent regulator of chromatin subfamily A member 5 (SMARCA5). HOXA1 is a known proto-oncogene, and it also plays an important role in embryonic development. The direct targeting of the miR-99 family to two candidate binding sequences located in the HOXA1 mRNA was confirmed using a luciferase reporter gene assay and a ribonucleoprotein-immunoprecipitation (RIP-IP) assay. Ectopic transfection of miR-99 family reduced the expression of HOXA1, which, in consequence, down-regulated the expression of its downstream gene (i.e., Bcl-2) and led to reduced proliferation and cell migration, as well as enhanced apoptosis. In summary, we identified a number of high-confidence miR-99 family target genes, including proto-oncogene HOXA1, which may play an important role in regulating epithelial cell proliferation and migration during physiological disease processes, such as dermal wound healing and tumorigenesis.

## Introduction

MicroRNAs are a class of small non-coding RNAs of approximately 22 nucleotides in length that are endogenously expressed in mammalian cells. MicroRNAs are not directly involved in protein coding, but are able to control the expression of their target genes at post-transcriptional levels by facilitating mRNA degradation and/or repressing translation. MicroRNAs have been shown to regulate many developmental and physiological processes, such as wound healing, as well as a number of disease processes, including cancer.

The miR-99 family is one of the evolutionarily most ancient microRNA families whose origin dates back before the bilaterian ancestor [1]–[3]. All available 88 mature miR-99 family microRNA sequences from miRBase (from 58 different species) have an identical seed region (which is thought to be the major determinant of target specificity), and as such, it is believed that they bind similar groups of targets. There are 3 members of the miR-99 family in humans (miR-99a, miR-99b and miR-100) located on distinct chromosomal regions in human genome (Chr 21, Chr 19, and Chr 11, respectively), and their mature microRNA sequences are identical to that from mouse. Early in animal evolution, miR-99 was active in a small population of cells located at the anterior anatomical sites, such as the foregut (digestive opening), and appears to play a role in developmental timing and maintaining tissue identity [3]. This is comparable with the fate of important transcriptional regulator genes, such as the evolutionarily conserved Homeobox (HOX) gene clusters. It became apparent recently that miR-99 family also expressed in several adult tissues (and cells) and play important roles in physiological and disease processes. Using dermal wound healing and oral squamous cell carcinoma as independent model systems, two of our recent studies showed that the miR-99 family members play an important role in regulating proliferation and migration of skin and oral mucosa epithelial cells, respectively [4], [5]. Our dermal wound healing study showed that the miR-99 family members are co-regulated (exhibit similar expression patterns) during the wound healing process, and the downregulation of miR-99 family contributes to re-epithelialization of the wound by regulating cell proliferation, apoptosis and cell migration [4]. It is worth noting that similar downregulation pattern of miR-99 family members was also observed during fin regeneration in zebrafish [6]. Our oral squamous cell carcinoma paper showed that down-regulation of miR-99 family members is one of the most consistently observed microRNA alterations in oral/head and neck cancer, and the reduction in miR-99 family expression is associated with enhanced cell proliferation and cell migration [5]. The deregulation of miR-99 family members is also a frequent event in several types of cancer of epithelial origin and they appear to play similar roles in the tumorigenesis of these cancers [7]–[10].

Most of the recent functional studies have linked the miR-99 family to the regulation of AKT/mTOR signaling pathway [4], [5], [8], [11]–[13]. However, these studies often focus on a specific cancer type, and may only reflect a fraction of the biological attributes of this microRNA family. Consequently, it is possible that many functional miR-99 family target genes still remain to be identified. In this study, using combined genomic and bioinformatics approaches, we identified a panel of high confidence miR-99 family target. We also functionally assessed the role of selected target genes, including Homeobox A1 (HOXA1), in miR-99 family-mediated regulation of proliferation and cell migration in epithelial cells.

## Materials and Methods

### Cell Culture and Transfection

The human immortal skin keratinocyte cell line HaCaT [14] was maintained in high glucose DMEM medium (Gibco) supplemented with 10% FBS, 100 units/ml penicillin, and 100 µg/ml streptomycin (Invitrogen). The human head and neck squamous carcinoma (HNSCC) cell lines (1386Ln [15], 1386Tu [15], 686Ln [16], 686Tu [16], SCC1 [17], SCC9 [18], SCC15 [18], SCC25 [18], UM1 [19], UM2 [19]) were maintained in DMEM/F12 medium (Gibco) supplemented with 10% FBS, 100 units/ml penicillin, and 100 µg/ml streptomycin (Invitrogen). All cells were maintained in a humidified incubator containing 5% CO_2_ at 37°C. For functional analysis, hsa-miR-99a, hsa-miR-99b, hsa-miR-100 and non-targeting microRNA mimic (Dharmacon), and gene specific siRNAs for mTOR, HOXA1, CTDSPL, NMT1, TMEM30A, SMARCA5 and Bcl-2 (Santa Cruz Biotechnology) were transfected into the cells using DharmaFECT Transfection Reagent 1 as described previously [20], [21].

### Microarray Analysis

The 1386Ln and HaCaT cells were transfected with miR-100 mimic and non-targeting miRNA mimic as described above, and the total RNA was isolated using miRNeasy Mini kit (Qiagen), and labeled and hybridized to the Affymetrix GeneChip Human Gene 1.0 ST arrays according to the standard protocol. The arrays were scanned with a GeneChip Scanner 3000. The scanned array images were processed with GeneChip Operating software (GCOS). The microarray data were pre-processed using Robust Multi-array Analysis (RMA) [22]. The microarray dataset has been submitted to ArrayExpress Archive (accession number: E-MTAB-1876). Existing microarray dataset on C4-2 prostate cancer cells treated with either miR-99a or control that was generated by Sun et al., 2011 [7] was downloaded from Gene Expression Omnibus (GEO accession GSE26332), and processed using RMA. Additional differential expression datasets on SCC29 oral cancer cells transfected with miR-100 or control, and 4T1 murine mammary tumor cells transfected with miR-100 or control were extracted from reports by Henson et al., 2009 [23] and Gebeshuber et al., 2012 [24], respectively.

### MicroRNA target prediction

The candidate targets of the miR-99 family were identified using the comparative analysis function of the miRWalk [25], which contains a collection of 10 bioinformatics tools, including DIANAmT, miRanda, miRDB, miRWalk, RNAhybrid, PicTar (4-way), PicTar (5-way), PITA, RNA22, TargetScan5.1. In addition, MicroCosm 5.0 and TargetScanHuman 6.2 were also used for predicting the miR-99 family targets. For our study, genes that were predicted by at least one method were defined as potential miR-99 family targets. The base-pairing and the minimum free energy (mfe) for the binding of microRNA to its targeting sequences were predicted using the RNAhybrid program [26].

### Quantitative RT-PCR Analysis

The total RNA from cultured cells was isolated using miRNeasy Mini kit (Qiagen). The total RNA samples from mouse embryos of different stages (7-, 11-, 15-, 17-day embryo), and from adult brain, eye, and salivary gland tissues were obtained from Clontech. The relative levels of miR-99a, miR-99b and miR-100 were determined by TaqMan microRNA assays (Applied Biosystems) as previously described [4], [5]. The relative mRNA levels of mTOR, HOXA1, CTDSPL, NMT1, TMEM30A and SMARCA5 were determined by quantitative two-step RT-PCR assay with gene specific primer sets (Origene) as described before [4], [5]. The relative microRNA and mRNA levels were computed using the 2^-delta delta Ct^ analysis method, where U6 and beta-actin were used as internal controls, respectively.

### Cell proliferation, migration and apoptosis assays

Cell proliferation was measured using the MTT [3-(4,5-dimethylthiazol-2-yl)-2,5-diphenyl-2H-tetrazolium bromide] assay as described previously [4], [27]. In brief, cells were grown in 96-well plates to about 70% confluence and transiently transfected with the desired microRNA mimics or siRNAs. At 48 h post transfection, medium in each well was replaced by 100 μl of fresh serum free medium with 0.5 g/L MTT. After incubation at 37°C for 4 h, the MTT medium was aspirated out and 50 μl of DMSO was added to each well. After incubation at 37°C for another 10 min, the absorbance value of each well was measured using a plate reader at a wavelength of 540 nm. Cell migration was measured using a trans-well assay as described previously [4], [28] using BD BioCoat Control Cell Culture Inserts (containing an 8.0 *μ*m PET Membrane without matrix). In brief, approximately 5×10^4^ cells were seeded in the upper chambers, and culture medium with 10% FBS was added to the lower chambers. After 48-h incubation, cells that migrated to the reverse side of inserts were stained with Diff-Quik stain kit (Polysciences, Inc., Warrington, PA) and quantified. The apoptosis was measured using the Annexin V-FITC Apoptosis Detection Kit (Invitrogen) as previously described [4], [27]. In brief, cells were grown in 6-well plates to about 70% confluence and transiently transfected with the desired microRNA mimics or siRNAs. The cells were digested and collected after 48 h post-transfection, and washed with PBS twice, then resuspended in 1×Binding Buffer, and 5 μl of Annexin FITC Conjugate, and 10 μl of propidium iodide solution were added to each cell suspension, separately. The stained cells (1×10^5^) were then analyzed with a flow cytometer (FACScalibur, Becton-Dickinson).

### Western-blot analysis

Western blots were performed as described previously [29] using antibodies specific for mTOR, Bcl-2 (Cell Signaling), HOXA1 and beta-actin (Sigma-Aldrich) and an immuno-star HRP substrate Kit (Bio-RAD).

### Dual-Luciferase reporter assay

The luciferase reporter gene constructs (pGL-TS1 and pGL-TS2) were created by cloning a 57-bp fragment from the coding region (position 409–465 of the HOXA1 mRNA sequence NM_005522, containing the miR-99 family targeting site TS1) and a 54-bp fragment from the 3′-UTR (position 2155–2208 of the HOXA1 mRNA sequence NM_005522, containing the miR-99 family targeting site TS2) into the Xba I site of the pGL3-Control firefly luciferase reporter vector (Promega) as described previously [30]. The corresponding mutant constructs (pGL-TS1m and pGL-TS2m) were created by replacing the seed regions (positions 2–8) of the miR-99 family binding sites with 5′-TTTTTTT-3′. All constructs were verified by sequencing. The reporter constructs and the pRL-TK vector (Promega) were co-transfected using Lipofectamine 2000 (Invitrogen). The luciferase activities were then determined as described previously [20] using a GloMax 20/20 luminometer (Promega). Experiments were performed in quadruplicate.

### Ribonucleoprotein-IP (RIP-IP) assay

RIP-IP assays were performed as described previously [4], [31]. Briefly, cells were co-transfected with a pIRESneo-FLAG/HA-Ago2 expression vector (Addgene plasmid 10822, Addgene Inc.) and miR-100 mimic or non-targeting microRNA mimic (Dharmacon). 48 h after transfection, cells were washed and lysed in radioimmune precipitation buffer (Sigma) containing 10% proteinase inhibitor cocktail (Sigma), 1 mM PMSF (Fluka), and 100 units/ml SUPERase·In (Ambion). The samples were then subjected to centrifugation for 30 min at 14,000 rpm, and the supernatants were collected. A fraction of the whole cell lysate was saved for RNA isolation, and the remaining lysate was subjected to immunoprecipitation (IP) using anti-FLAG M2 affinity gel (Sigma). RNA from the whole cell lysate and the RIP-IP fraction was extracted with QIAzol and purified by miRNeasy mini kit (Qiagen). The relative mRNA level of the HOXA1 was determined using a quantitative two-step RT-PCR as described. The relative enrichment of mRNA in the RIP-IP fractions was computed based on the ratio of relative mRNA levels in the RIP-IP fractions and the relative mRNA levels in the whole cell lysates as described previously [4], [31].

### Statistical analysis

Data was analyzed using the Statistical Package for Social Science (SPSS), version 17.0. Student's t-test was used to compare differences between groups. Fisher's exact test was used to test the enrichment of predicted microRNA genes in the gene list. Pearson's correlation coefficient was computed for examining the relationship between the expression of microRNA and their target genes. For all analyses, p<0.05 was considered statistically significant.

## Results and Discussion

MicroRNA can have multiple targets. We performed microarray-based differential expression analysis on human 1386Ln and HaCaT cells transfected with miR-100 mimic and negative control mimic. We also obtained 2 existing microarray datasets with similar study design (microarray analysis on C4-2 human prostate cancer cells transfected with miR-99a or control [7], and SCC29 human oral cancer cells transfected with miR-100 or control [23]). The 5^th^ microarray dataset is based on a mouse cell line (4T1 murine mammary tumor cells transfected with miR-100 or control [24]). Since human and mouse miR-99 family microRNA sequences are identical (and consequently, may have similar targets), we also included this mouse cell line-based dataset in our analysis. All 5 cell lines used in these microarray experiments are cells of epithelial origin. As shown in **Table S1**, there are 266 genes that were down-regulated by miR-99 family members (miR-100 or miR-99a) in at least 2 microarray experiments. The top 24 candidates (down-regulated in at least 3 microarray experiments) are listed in **Table 1**.

**Table 1:** miR-100 target genes identified in epithelial cells by microarray analysis and bioinformatics prediction.

Gene a	Genes down-regulated by miR-100 treatment (microarray analysis)					Bioinformatics predicted targets of miR-100 f
	HaCaT b	1386Ln b	C4-2 c	SCC29 d	4T1 e	
**CTDSPL**	1	1	1	1		7
**NMT1**	1	1	1	1		2
**TMEM30A**	1	1	1	1		4
ATP6AP1		1	1	1		4
BMPR2	1		1		1	4
CDK6	1	1	1			1
DNAJB4	1	1	1			2
FABP5	1		1		1	0
HIPK3	1	1	1			0
**HOXA1**	1		1	1		10
KBTBD8	1		1	1		8
KCTD10		1	1	1		3
LRRC8B	1	1	1			1
MMP13	1			1	1	0
**mTOR**	1	1	1			12
RAB15	1		1		1	2
REEP3	1	1	1			0
SLC39A6	1	1	1			1
**SMARCA5**	1	1	1			11
SMEK2	1	1	1			5
SNX9	1		1	1		4
TTC30A		1	1	1		6
VLDLR	1	1			1	7
ZDHHC18	1	1		1		5

a Gene names in bold font were genes that down-regulated in 4 out of 5 microRNA transfection experiments, or down-regulated in 3 out of 5 microRNA transfection experiments and predicted to be miR-100 targets by 10 out of 12 bioinformatics tools tested.

b Human skin keratinocyte HaCaT and head and neck squamous cell carcinoma cell 1386Ln were treated with either miR-100 or control mimic and differential expression analysis was carried out using Affymetrix GeneChip HuGene 1.0 ST arrays. The data was processed using Robust Multi-array Analysis (RMA), and the down-regulated gene was defined as a gene with a microRNA-induced expressional change equal or less than the known target gene mTOR (fold difference  = 0.88 and 0.67, respectively for HaCaT and 1386Ln cells).

c Microarray data on C4-2 prostate cancer cells treated with either miR-99a or control (GEO accession GSE26332) [7], and processed using Robust Multi-array Analysis (RMA). The down-regulated gene was defined as a gene with a microRNA-induced expressional change equal or less than the known target gene mTOR (fold difference  = 0.67).

d Data from Henson et al 2009 [23].

e Data from Gebeshuber et al 2012 [24].

f The candidate targets of miR-100 were predicted using a collection of 12 bioinformatics tools, including DIANAmT, miRanda, microCosm, miRDB, miRWalk, RNAhybrid, PicTar (4-way), PicTar (5-way), PITA, RNA22, TargetScan5, and TargetScanHuman 6.2. The number of bioinformatics tools (out of a total of 12 tools tested here) that predict a gene to be miR-100 target was presented.

These microarray-based experiments measure the differential expression of mRNA levels, and are only sensitive to the targets that are regulated by microRNA mediated degradation, but not to the targets that are regulated by microRNA mediated translational inhibition. We anticipate that a portion of true miR-100 targets will not be detected by our approach. In fact, IGF1R, a gene that was previously shown to be inhibited by miR-100 through translation inhibition [4], [32], was not detected in our microarray experiment. Nevertheless, our study identified a panel of genes regulated by miR-100, including the experimentally-confirmed miR-100 target gene, mammalian Target of rapamycin (mTOR), Homeobox A1 (HOXA1), CTD small phosphatase-like (CTDSPL), and SWI/SNF-related matrix-associated actin-dependent regulator of chromatin subfamily A member 5 (SMARCA5). As a complementary approach to our microarray analysis, we also carried out a bioinformatics-based target prediction using 12 different sequence-based microRNA target prediction algorithms (DIANAmT, miRanda, microCosm, miRDB, miRWalk, RNAhybrid, PicTar (4-way), PicTar (5-way), PITA, RNA22, TargetScan5.1, and TargetScanHuman6.2) to refine our list of target genes for miR-100. Each bioinformatics tool utilizes a different model to define targeting sequences that are associated with functionality. Consequently, the predictions will differ when applied to the same microRNAs, with each method having different levels of coverage and false positive prediction [33]. These differences reflect the varying biological attributes that each mathematical model highlights. As shown in **Table S1**, 81 out of these 266 down-regulated transcripts are potential direct targets for miR-100. Among the top 24 candidates (down-regulated in at least 3 microarray experiments), 20 of them are potential direct target genes of miR-100 (**Table 1**). As such, bioinformaticsly predicted miR-100 target genes are significantly enriched in the top 24 candidates (Fisher's exact test, p<0.0001).

**Figure 1:**
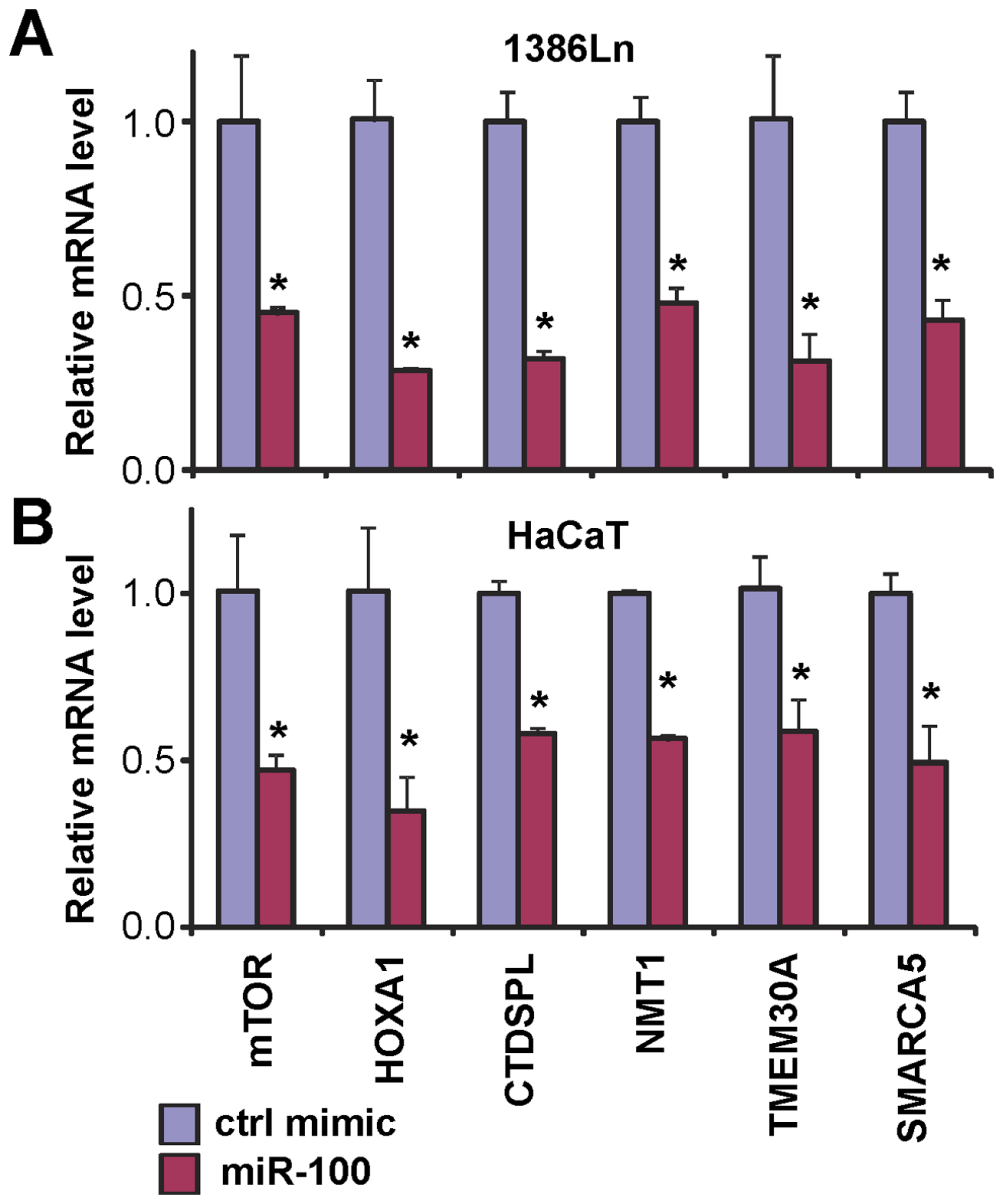
MiR-100-mediated down-regulation of its target genes. The miR-100 mimic and negative control microRNA were introduced into the 1386Ln (**A**) and HaCaT cells (**B**). qRT-PCR was performed to assess the expression of mTOR, HOXA1, CTDSPL, NMT1, TMEM30A and SMARCA5. Data represents at least 3 independent triplicate experiments with similar results. * indicates p<0.05.

We selected 6 high-confidence targets (down-regulated in at least 3 microarray experiments and consistently predicted by 10 out of 12 bioinformatics methods, or down-regulated in 4 out of 5 microarray experiments) as candidates for further confirmation experiments and functional analysis. These high-confidence target genes are mTOR, HOXA1, CTDSPL, NMT1, TMEM30A, and SMARCA5. Among these genes, mTOR, CTDSPL and SMARCA5 have been previously identified and experimentally confirmed as functional targets for miR-99 family members [5], [7], [11], [34], [35]. HOXA1 is a known oncogene [36]–[40], and NMT1 also plays a role in regulating tumor cell proliferation and apoptosis [41], [42]. TMEM30A plays a major role in cell polarity control and microtubule regulation, and is also involved in cell migration [43]. As shown in **Figure 1**, the miR-100-mediated down-regulation of these 6 candidate genes in 1386Ln and HaCaT cells was validated by quantitative RT-PCR analysis.

**Figure 2:**
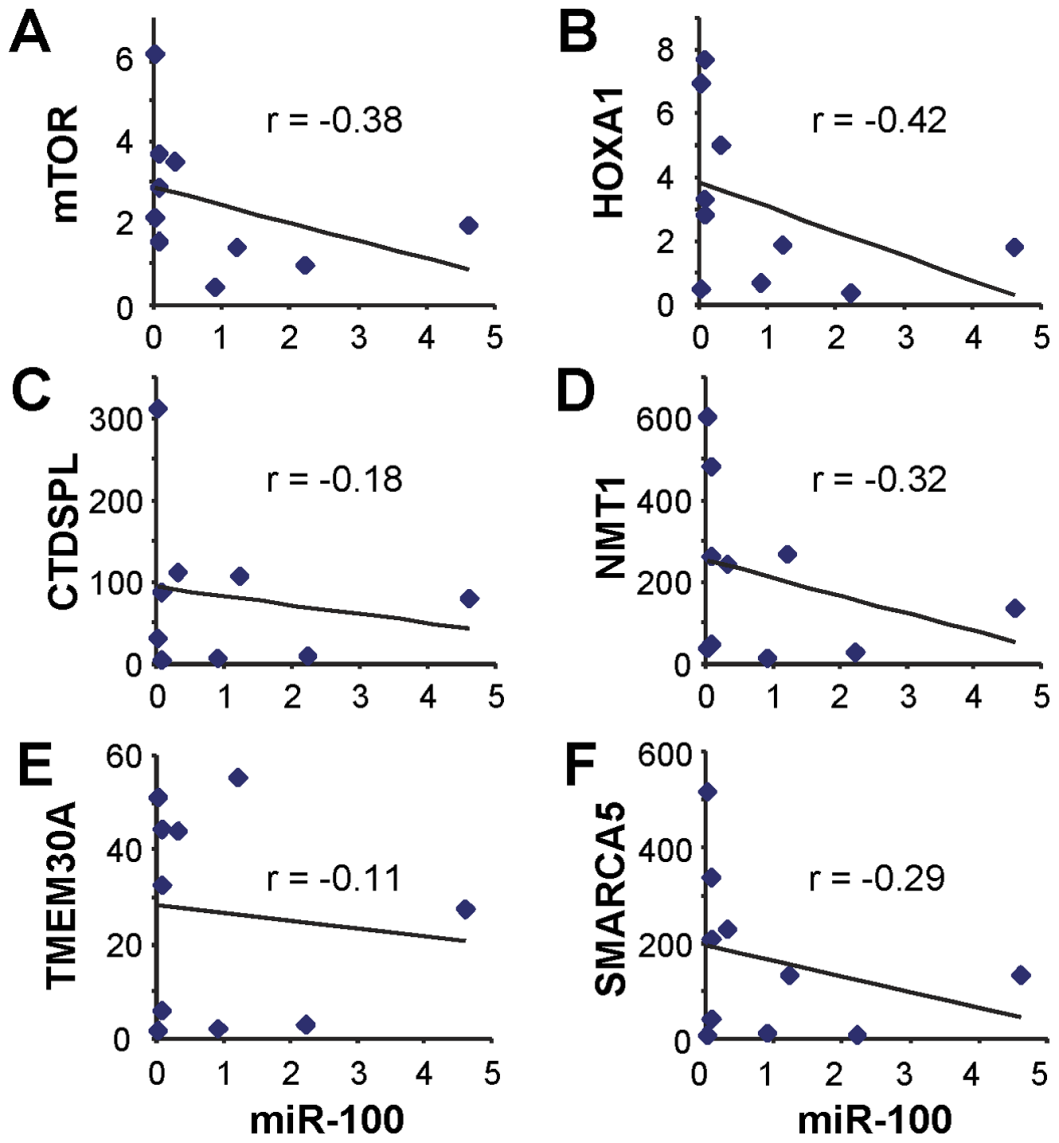
Correlation of miR-100 level and the expression of its target genes in HNSCC. The level of miR-100 and the expression of mTOR, HOXA1, CTDSPL, NMT1, TMEM30A and SMARCA5 were assessed by qRT-PCR in 10 HNSCC cell lines. Potential correlation of the miR-100 level with the expression of mTOR (**A**), HOXA1 (**B**), CTDSPL (**C**), NMT1 (**D**), TMEM30A (**E**), and SMARCA5 (**F**) was assessed, and the Pearson's correlation coefficient (r) was calculated.

To further evaluate these candidate miR-100 target genes, we examined the relationship between the miR-100 level and the expression of these 6 target genes in 10 HNSCC cell lines (**Figure 2**). Among these 6 genes, mTOR (**Figure 2A**), CTDSPL (**Figure 2C**), and SMARCA5 (**Figure 2F**) are experimentally confirmed miR-100 target genes by previous studies [5], [7], [11], [34], [35], and their expression exhibited apparent inverse correlations with the miR-100 level in the HNSCC cell lines (Pearson's correlation coefficient  = −0.38, −0.18 and −0.29, respectively). Using these 3 known target genes as guidance, it appears that the expression levels of HOXA1 and NMT1 are also inversely correlated with the level of miR-100 in the HNSCC cell lines (**Figure 2B and 2D**, Pearson's correlation coefficient  = −0.42 and −0.32, respectively). No apparent correlation between the TMEM30A and miR-100 was observed (**Figure 2E**).

**Figure 3:**
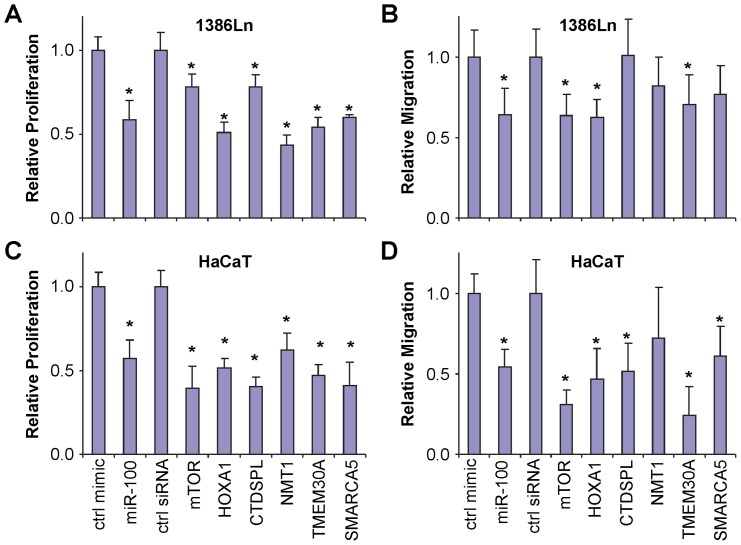
The effect of miR-100 target genes on proliferation and cell migration. 1386Ln (**A** and **B**) and HaCaT cells (**C** and **D**) were transfected with either negative control microRNA, miR-100 mimic, negative control siRNA, or specific siRNAs against mTOR, HOXA1, CTDSPL, NMT1, TMEM30A or SMARCA5. Proliferation (**A** and **C**) and cell migration (**B** and **D**) were measured as described in the Material and Methods section. Data represents at least 3 independent triplicate experiments with similar results. * indicates p<0.05.

The miR-99 family members (miR-99a/b and miR-100) have been shown to regulate cell proliferation and cell migration in several types of cancer of epithelial origin [5], [7], [9], and during dermal wound healing [4]. As such, we chose to further explore the functional role of these 6 miR-99 family target genes in our study. As shown in **Figure 3A and 3C**, ectopic transfection of miR-100 mimic to 1386Ln and HaCaT cells led to a statistically significant down-regulation in cell proliferation as compared to cells treated with control mimic (measured by MTT assay). When cells were treated with siRNAs specific to mTOR, HOXA1, CTDSPL, NMT1, TMEM30A and SMARCA5, statistically significant down-regulation of these genes were confirmed as compared to cells treated with control siRNA (**Figure S1**). These siRNA-mediated gene expression changes were accompanied by statistically significant down-regulation in cell proliferation (**Figure 3A and 3C**). As shown in **Figure 3B and 3D**, ectopic transfection of miR-100 mimic to 1386Ln and HaCaT cells led to a statistically significant down-regulation in cell migration as compared to the cells treated with control mimic (measured by trans-well assay). Statistically significant down-regulation in cell migration was also observed in 1386LN cells that were treated with siRNAs specific to mTOR, HOXA1, and TMEM30A, and in HaCaT cells treated with siRNAs specific to mTOR, HOXA1, CTDSPL, TMEM30A and SMARCA5, as compare to cells treated with control siRNA. These results, together with the expressional correlation test in HNSCC cell lines, suggest that mTOR, HOXA1 and SMARCA5 appear to be the best functional candidate target genes for the miR-99 family in cells of epithelial origin.

**Figure 4:**
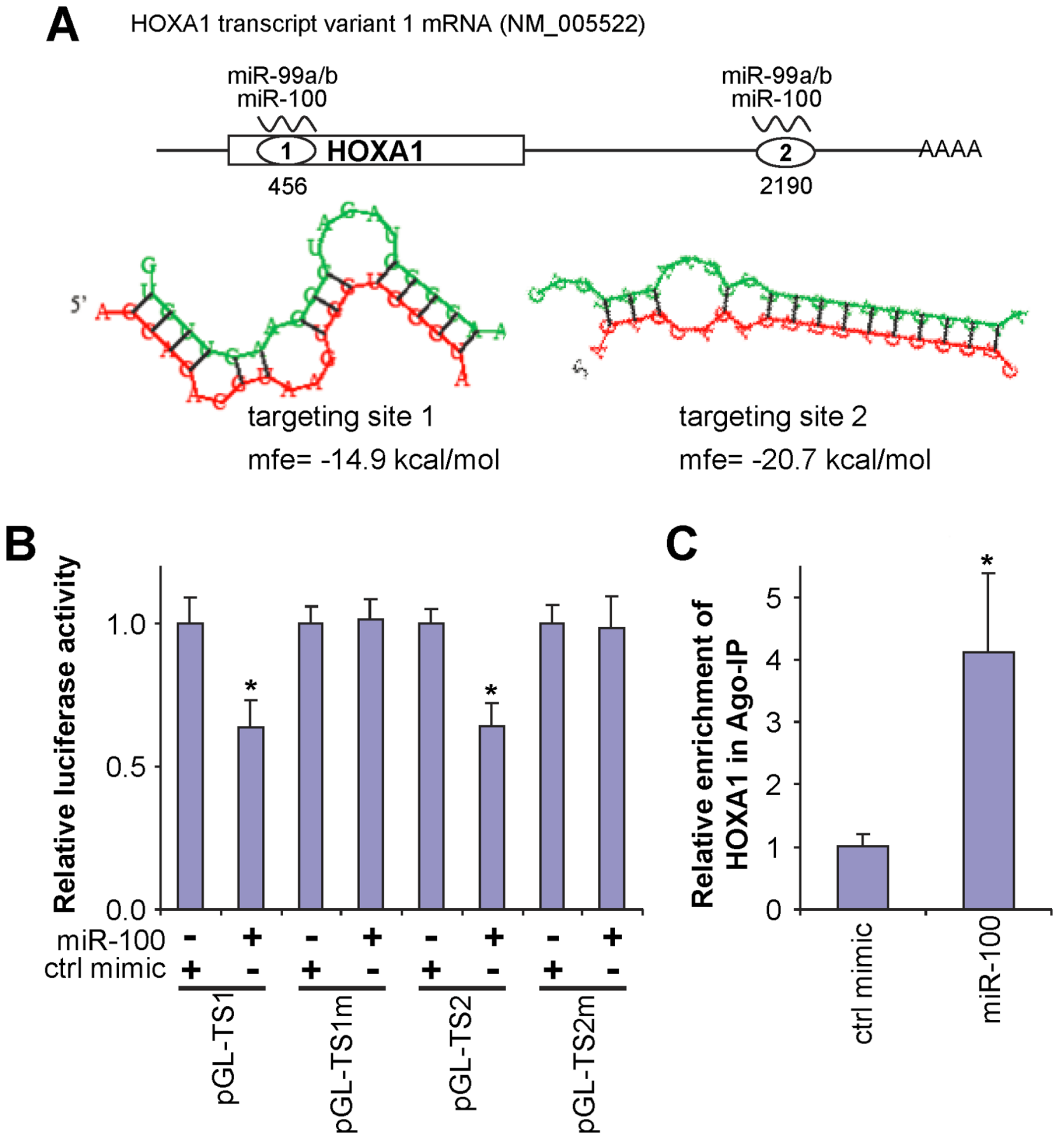
MiR-100 direct targeting HOXA1 mRNA. (**A**) Two predicted miR-99 family targeting sequences (TS1 and TS2) are located in the coding region and the 3′-UTR of HOXA1 mRNA, respectively. The base-pairing (green: microRNA sequence; red: mRNA sequence) and the minimum free energy (mfe) for the binding of miR-100 to the targeting sequences were predicted using the RNAhybrid program [26]. (**B**) Dual luciferase reporter assays were performed to test the interaction of miR-100 and its targeting sequences in the HOXA1 mRNA using constructs containing the predicted targeting sequences (pGL-TS1 and pGL-TS2) and mutated targeting sequences (pGL-TS1m and pGL-TS2m) cloned into the 3′-UTR of the reporter gene. (**C**) RIP-IP assays were performed to co-IP the Ago2 complexes from cells transfected with either miR-100 mimic or negative control mimic. qRT-PCR assays were performed on RNA samples isolated from the Ago2 co-IP fractions to measure the relative enrichment of the HOXA1 mRNA. Data represent at least 3 independent experiments with similar results. *: p<0.05.

While the targeting sequences for miR-99 members on mTOR and SMARCA5 mRNAs have been previously identified and experimentally confirmed [5], [7], [11], [34], the direct interaction of the miR-99 family and HOXA1 mRNA has not been defined. Bioinformatics analysis revealed that there are 2 miR-99 family targeting sites in the HOXA1 mRNA, one located in the coding region and a second located in the 3′-UTR (**Figure 4A**). The HOXA1 gene has 2 known transcript variants, resulting from alternative utilization of 5′ exons (**Figure S2**). The transcript variant 2 has a truncated coding region and appears to be nonfunctional. The first miR-99 family targeting site is not present in the transcript variant 2. Both variants have identical 3′-UTR, and as such, they both contain the second miR-99 family targeting site. To test whether the miR-99 family directly interacts with these predicted targeting sites in HOXA1 mRNA, dual luciferase reporter assays were performed using constructs containing these targeting sites (**Figure 4B**). When cells were transfected with miR-100, the luciferase activities of the constructs containing both targeting sites (pGL-TS1 and pGL-TS2) were significantly reduced as compared to the cells transfected with negative control. When the seed regions of these two targeting sites were mutated (pGL-TS1m and pGL-TS2m), the effect of miR-100 on the luciferase activity was abolished. These results confirmed that miR-100 directly interacts with these targeting sites in HOXA1 mRNA. Mature microRNAs form stable complexes with Argonaute proteins (such as Ago2), the core of the RNAi-induced silencing complex (RISC). The microRNA then directs RISC to bind to the mRNA molecules containing specific targeting sequences, and results in translational repression and/or enhanced mRNA degradation. To further confirm that miR-100 directly interacts with HOXA1 mRNA, we tested the miR-100-mediated binding of RISC to HOXA1 mRNA using an Ago2-based ribonucleoprotein-IP assay (RIP-IP). As shown in **Figure 4C**, the Ago2 co-IP fractions from cells treated with miR-100 mimic were significantly enriched in HOXA1 mRNA as compared to cells treated with control mimic. It is worth noting that the expressions of HOXA3 and HOXA10 were also down-regulated by the miR-99 family in HaCaT and C4-2 cells, and in C4-2 and SCC29 cells, respectively (**Table S1**). Two miR-99 family targeting sites were predicted, located in the 5′-UTR and coding region of HOXA3 mRNA, respectively (**Figure S3**). No targeting sequence was predicted in HOXA10. It is possible that the miR-99 family indirectly regulates HOXA10 by targeting factors that control the expression of this gene. Alternatively, this HOX gene may be regulated by the miR-99 family through noncanonical targeting sequences. Additional studies are needed to explore the mechanisms that contribute to miR-99-mediated expressional change of additional HOX genes.

**Figure 5:**
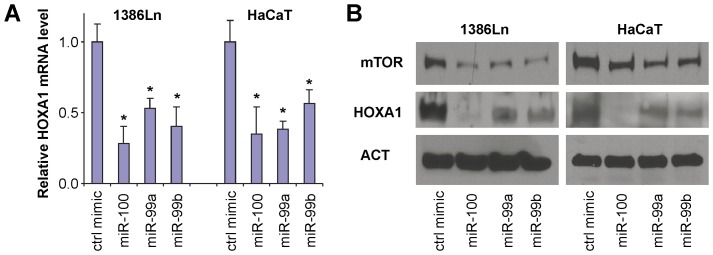
The effect of miR-99 family members on HOXA1 expression. 1386Ln and HaCaT cells were treated with mimics for miR-99 family members (miR-100, miR-99a, miR-99b), or negative control microRNA mimic. The effects of miR-99 family members on HOXA1 gene expression were examined by qRT-PCR (**A**) and Western blot analysis (**B**).

In addition to miR-100, we also examined the effect of other members of miR-99 family (miR-99a and miR-99b) on the HOXA1 expression. As shown in **Figure 5**, ectopic transfection of miR-99a, miR-99b, and miR-100 mimic to 1386Ln and HaCaT cells led to a statistically significant down-regulation of HOXA1 gene expression at the mRNA level (**Figure 5A**) and at the protein level (**Figure 5B**). In contrast, knockdown of miR-99 family members using a LNA inhibitor enhanced the expression of HOXA1 (**Figure S4**). These results, together with the luciferase reporter assay and RIP-IP assay, provide solid evidence supporting that members of the miR-99 family down-regulate the HOXA1 expression by directly interacting with HOXA1 mRNA. As such, our results demonstrate that in addition to mTOR and SMARCA5, HOXA1 is another experimentally confirmed functional target gene of miR-99 family members.

**Figure 6:**
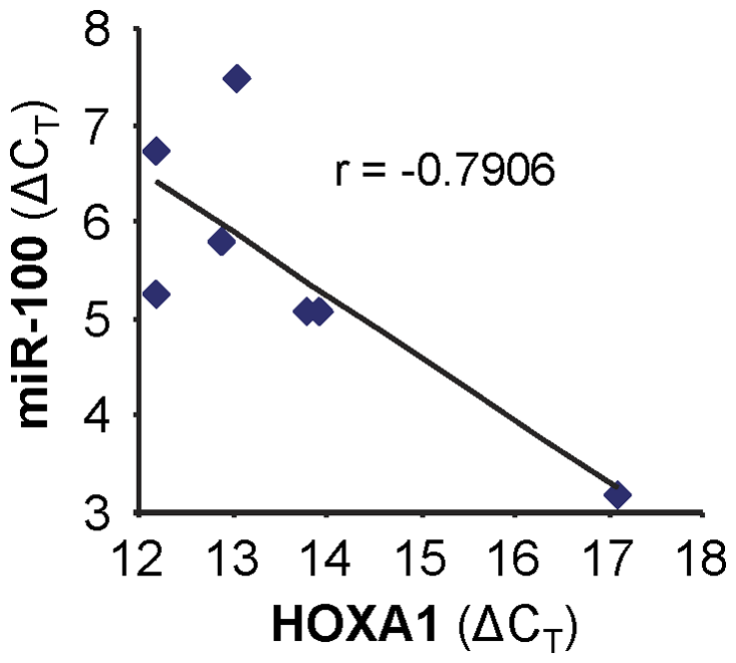
Correlation of miR-100 and HOXA1 levels in various tissues. The levels of miR-100 and HOXA1 were determined in mouse embryos of different stages (7-, 11-, 15-, 17-day embryo), and adult tissues of anterior anatomical location (brain, eye, and salivary gland) by qRT-PCR. An inverse correlation between miR-100 and HOXA1 levels was observed (Pearson's correlation coefficient  = −0.79, p = 0.03).

The conserved family of HOX transcription factors is critically involved in patterning the body plan of bilaterian embryos by controlling multiple morphogenetic and organogenetic processes during development [44]–[47]. In mammals, there are 39 HOX genes present in 4 paralogous gene clusters named HOXA, HOXB, HOXC, and HOXD. During development, the expression of HOX genes is under a strict temporospatial control in a manner that the 3′ HOX genes (e.g., HOXA1) are expressed prior to the 5′ genes (e.g., HOXA9) within a given clusters. This expression pattern is critical for ensuring segmental identity and morphology on the anteroposterior axis (e.g., 3′ HOX genes are associated with the development of anterior tissues and 5′ HOX genes are associated with posterior tissues). HOXA1 is the most 3′ HOX gene in cluster A, and one of the earliest HOX genes to be expressed during embryonic development. It is also the first genes expressed in the central nervous system, and accordingly, plays a critical role in brain and head development. HOXA1 is also expressed in several adult tissues, where they perform important roles in maintaining homeostasis. We examined the level of miR-100 and HOXA1 expression in mouse embryos of different stages, and adult tissues of anterior anatomical location (brain, eye, and salivary gland). As shown in **Figure 6**, an apparent inverse correlation was observed between miR-100 and HOXA1 levels (r = −0.79, p = 0.03). This further supported our conclusion that HOXA1 is a target gene of miR-100, and also suggested that the interaction between miR-100 and HOXA1 may play a role in development. It is worth noting that HOXA1 (and other HOX family genes) is also controlled by other microRNAs, including the miR-10 and miR-196 families which reside within the HOX chromosomal clusters [48]–[51]. As such, microRNA-mediated post-transcriptional regulation provides another layer of control to the strict temporospatial expression pattern of the HOX gene family.

**Figure 7:**
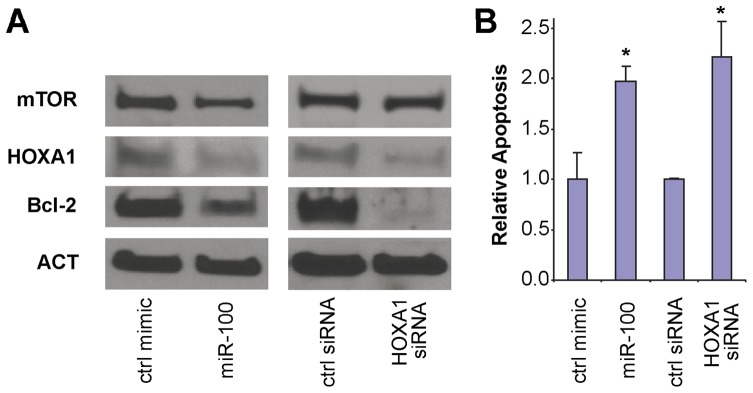
The effect of miR-100 on Bcl-2 expression and apoptosis. 1386Ln cells were treated with either miR-100 mimic, negative control microRNA, HOXA1 siRNA or control siRNA. The expression of mTOR, HOXA1, and Bcl-2 were assessed by Western blot analysis (**A**). Apoptosis was measured by flow cytometry (**B**). Data represent at least 3 independent experiments with similar results. *: p<0.05.

In addition to its role in development, HOXA1 is an established oncogene [36]–[40], and its over-expression has been observed in several types of solid tumor, including oral/head and neck cancer [52], [53]. The HOXA1-stimulated oncogenic transformation is mediated by transcriptional up-regulation of a number of down-stream genes, such as the anti-apoptotic gene Bcl-2 [36], [54]. As shown in **Figure 7A**, while ectopic transfection of miR-100 mimic to 1386Ln cells led to the down-regulation of mTOR, HOXA1 and Bcl-2 expression as compare to control mimic treated cells, the HOXA1 siRNA treated 1386Ln cells exhibited reduced expression of HOXA1 and Bcl-2 and no apparent change in mTOR level. The Bcl-2 expression in HaCaT was undetectable, which is consistent with previous observation [55]. As shown in **Figure 7B**, both miR-100 and HOXA1 siRNA treated 1386Ln cells exhibited statistically significant increases in apoptosis as compare to control mimic or control siRNA treated cells, respectively. This is in agreement with previous observation showing that HOXA1 promotes cell survival by up-regulating Bcl-2 [36], [54].

In summary, we identified a panel of high confidence miR-99 family target genes, including previously reported target genes mTOR, CTDSPL and SMARCA5, and novel candidates HOXA1, CTDSPL, NMT1, and TMEM30A. Our data suggests that the miR-99 family regulates cell proliferation, migration and apoptosis in part by regulating the expression of HOXA1. We further demonstrated that the miR-99 family regulates HOXA1 expression by directly interacting with 2 target sites in HOXA1 mRNA. Further studies are required to explore the role of miR-99 family-regulated HOXA1 expression in development, tumorigenesis, and wound healing.

## Supporting Information

Figure S1 **siRNA-mediated down-regulation of mTOR, HOXA1, CTDSPL, NMT1, TMEM30A and SMARCA5 gene expression.** 1386Ln (**A**) and HaCaT cells (**B**) were transfected with either negative control siRNA, or specific siRNAs against mTOR, HOXA1, CTDSPL, NMT1, TMEM30A or SMARCA5. The relative mRNA levels of these genes were measured by qRT-PCR. * indicates p<0.05.
(PPT)

Figure S2 **Predicted hsa-miR-99 family targeting sites on HOXA1 mRNAs.** The predicted miR-99 family targeting sites on the mRNA sequences of (**A**) HOXA1 transcript variant 1 (NM_005522) and (**B**) HOXA1 transcript variant 2 (NM_153620). Two predicted targeting sites were identified in the transcript variant 1 of the HOXA1 gene, targeting site 1 located in the coding region and targeting site 2 located in the 3′-UTR, respectively. Only targeting site 2 was presented in the transcript variant 2 of the HOXA1 gene. The base-pairing (green: microRNA sequence; red: mRNA sequence) and the minimum free energy (mfe) for the binding of hsa-miR-100 to the targeting site 1 (**C**) and the targeting site 2 (**D**) were predicted using the RNAhybrid program [Krüger & Rehmsmeier: RNAhybrid: microRNA target prediction easy, fast and flexible. Nucleic Acids Res. 2006 Jul 1;34(Web Server issue):W451–4].
(PPT)

Figure S3 **Predicted hsa-miR-99 family targeting sites on HOXA3 mRNA.** (**A**) Two predicted targeting sites were predicted in the HOXA3 mRNA (NM_030661), located in the 5′-UTR and coding region, respectively. The base-pairing (green: microRNA sequence; red: mRNA sequence) and the minimum free energy (mfe) for the binding of hsa-miR-100 to the targeting site 1 (**B**) and the targeting site 2 (**C**) were predicted using the RNAhybrid program [Krüger & Rehmsmeier: RNAhybrid: microRNA target prediction easy, fast and flexible. Nucleic Acids Res. 2006 Jul 1;34(Web Server issue):W451–4].
(PPT)

Figure S4 **The effect of miR-99 family LNA inhibitor on HOXA1 expression.** 1386Ln and HaCaT cells were treated with LNA inhibitor for miR-99 family, or negative control LNA. The expression of HOXA1 gene was examined by qRT-PCR. *: p<0.05.
(PPT)

Table S1 **Genes that down-regulated by miR-99 family members.**
(XLS)

## References

[1] Hertel J, Bartschat S, Wintsche A, Otto C, Of The Bioinformatics Computer Lab TS, et al (2012) Evolution of the let-7 microRNA Family. RNA Biol 9: 231–241. 22617875 10.4161/rna.18974 PMC3384580

[2] Heimberg AM, Sempere LF, Moy VN, Donoghue PC, Peterson KJ (2008) MicroRNAs and the advent of vertebrate morphological complexity. Proc Natl Acad Sci U S A 105: 2946–2950. 18287013 10.1073/pnas.0712259105 PMC2268565

[3] Christodoulou F, Raible F, Tomer R, Simakov O, Trachana K, et al (2010) Ancient animal microRNAs and the evolution of tissue identity. Nature 463: 1084–1088. 20118916 10.1038/nature08744 PMC2981144

[4] Jin Y, Tymen SD, Chen D, Fang ZJ, Zhao Y, et al (2013) MicroRNA-99 Family Targets AKT/mTOR Signaling Pathway in Dermal Wound Healing. PLoS One 8: e64434. 23724047 10.1371/journal.pone.0064434 PMC3665798

[5] Chen Z, Jin Y, Yu D, Wang A, Mahjabeen I, et al (2012) Down-regulation of the microRNA-99 family members in head and neck squamous cell carcinoma. Oral Oncol 48: 686–691. 22425712 10.1016/j.oraloncology.2012.02.020 PMC3380146

[6] Yin VP, Thomson JM, Thummel R, Hyde DR, Hammond SM, et al (2008) Fgf-dependent depletion of microRNA-133 promotes appendage regeneration in zebrafish. Genes Dev 22: 728–733. 18347091 10.1101/gad.1641808 PMC2275425

[7] Sun D, Lee YS, Malhotra A, Kim HK, Matecic M, et al (2011) miR-99 family of MicroRNAs suppresses the expression of prostate-specific antigen and prostate cancer cell proliferation. Cancer Res 71: 1313–1324. 21212412 10.1158/0008-5472.CAN-10-1031 PMC3523179

[8] Sun J, Chen Z, Tan X, Zhou F, Tan F, et al (2013) MicroRNA-99a/100 promotes apoptosis by targeting mTOR in human esophageal squamous cell carcinoma. Med Oncol 30: 411. 23292834 10.1007/s12032-012-0411-9

[9] Li BH, Zhou JS, Ye F, Cheng XD, Zhou CY, et al (2011) Reduced miR-100 expression in cervical cancer and precursors and its carcinogenic effect through targeting PLK1 protein. Eur J Cancer 47: 2166–2174. 21636267 10.1016/j.ejca.2011.04.037

[10] Wong TS, Liu XB, Wong BY, Ng RW, Yuen AP, et al (2008) Mature miR-184 as Potential Oncogenic microRNA of Squamous Cell Carcinoma of Tongue. Clin Cancer Res 14: 2588–2592. 18451220 10.1158/1078-0432.CCR-07-0666

[11] Nagaraja AK, Creighton CJ, Yu Z, Zhu H, Gunaratne PH, et al (2010) A link between mir-100 and FRAP1/mTOR in clear cell ovarian cancer. Mol Endocrinol 24: 447–463. 20081105 10.1210/me.2009-0295 PMC2817607

[12] Tovar V, Alsinet C, Villanueva A, Hoshida Y, Chiang DY, et al (2010) IGF activation in a molecular subclass of hepatocellular carcinoma and pre-clinical efficacy of IGF-1R blockage. J Hepatol 52: 550–559. 20206398 10.1016/j.jhep.2010.01.015 PMC3662876

[13] Doghman M, El Wakil A, Cardinaud B, Thomas E, Wang J, et al (2010) Regulation of insulin-like growth factor-mammalian target of rapamycin signaling by microRNA in childhood adrenocortical tumors. Cancer Res 70: 4666–4675. 20484036 10.1158/0008-5472.CAN-09-3970 PMC2880211

[14] Boukamp P, Petrussevska RT, Breitkreutz D, Hornung J, Markham A, et al (1988) Normal keratinization in a spontaneously immortalized aneuploid human keratinocyte cell line. J Cell Biol 106: 761–771. 2450098 10.1083/jcb.106.3.761 PMC2115116

[15] Wreesmann VB, Wang D, Goberdhan A, Prasad M, Ngai I, et al (2004) Genetic abnormalities associated with nodal metastasis in head and neck cancer. Head Neck 26: 10–15. 14724901 10.1002/hed.10344

[16] Vigneswaran N, Wu J, Zacharias W (2003) Upregulation of cystatin M during the progression of oropharyngeal squamous cell carcinoma from primary tumor to metastasis. Oral Oncol 39: 559–568. 12798398 10.1016/s1368-8375(03)00038-1

[17] Krause CJ, Carey TE, Ott RW, Hurbis C, McClatchey KD, et al (1981) Human squamous cell carcinoma. Establishment and characterization of new permanent cell lines. Arch Otolaryngol 107: 703–710. 7295166 10.1001/archotol.1981.00790470051012

[18] Rheinwald JG, Beckett MA (1981) Tumorigenic keratinocyte lines requiring anchorage and fibroblast support cultures from human squamous cell carcinomas. Cancer Research 41: 1657–1663. 7214336

[19] Nakayama S, Sasaki A, Mese H, Alcalde RE, Matsumura T (1998) Establishment of high and low metastasis cell lines derived from a human tongue squamous cell carcinoma. Invasion Metastasis 18: 219–228. 10729767 10.1159/000024515

[20] Jiang L, Liu X, Kolokythas A, Yu J, Wang A, et al (2010) Downregulation of the Rho GTPase signaling pathway is involved in the microRNA-138-mediated inhibition of cell migration and invasion in tongue squamous cell carcinoma. Int J Cancer 127: 505–512. 20232393 10.1002/ijc.25320 PMC2885137

[21] Liu X, Jiang L, Wang A, Yu J, Shi F, et al (2009) MicroRNA-138 suppresses invasion and promotes apoptosis in head and neck squamous cell carcinoma cell lines. Cancer Lett 286: 217–222. 19540661 10.1016/j.canlet.2009.05.030 PMC2783372

[22] Irizarry RA, Bolstad BM, Collin F, Cope LM, Hobbs B, et al (2003) Summaries of Affymetrix GeneChip probe level data. Nucleic Acids Res 31: e15. 12582260 10.1093/nar/gng015 PMC150247

[23] Henson BJ, Bhattacharjee S, O'Dee DM, Feingold E, Gollin SM (2009) Decreased expression of miR-125b and miR-100 in oral cancer cells contributes to malignancy. Genes Chromosomes Cancer 48: 569–582. 19396866 10.1002/gcc.20666 PMC2726991

[24] Gebeshuber CA, Martinez J (2012) miR-100 suppresses IGF2 and inhibits breast tumorigenesis by interfering with proliferation and survival signaling. Oncogene. 10.1038/onc.2012.372 22926517

[25] Dweep H, Sticht C, Pandey P, Gretz N (2011) miRWalk–database: prediction of possible miRNA binding sites by “walking” the genes of three genomes. J Biomed Inform 44: 839–847. 21605702 10.1016/j.jbi.2011.05.002

[26] Kruger J, Rehmsmeier M (2006) RNAhybrid: microRNA target prediction easy, fast and flexible. Nucleic Acids Res 34: W451–454. 16845047 10.1093/nar/gkl243 PMC1538877

[27] Jiang L, Liu X, Chen Z, Jin Y, Heidbreder CE, et al (2010) MicroRNA-7 targets insulin-like growth factor 1 receptor (IGF1R) in tongue squamous cell carcinoma cells. Biochem J 432: 199–205. 20819078 10.1042/BJ20100859 PMC3130335

[28] Liu X, Wang C, Chen Z, Jin Y, Wang Y, et al (2011) MicroRNA-138 suppresses epithelial-mesenchymal transition in squamous cell carcinoma cell lines. Biochem J 440: 23–31. 21770894 10.1042/BJ20111006 PMC3331719

[29] Jiang L, Liu X, Kolokythas A, Yu J, Wang A, et al (2010) Down-regulation of the Rho GTPase signaling pathway is involved in the microRNA-138 mediated inhibition of cell migration and invasion in tongue squamous cell carcinoma. Int J Cancer 127: 505–512. 20232393 10.1002/ijc.25320 PMC2885137

[30] Jin Y, Chen Z, Liu X, Zhou X (2012) Evaluating the microRNA targeting sites by luciferase reporter gene assay. Methods Mol Biol 936: 117–127. 10.1007/978-1-62703-083-0_10 PMC3646406 23007504

[31] Jin Y, Wang C, Liu X, Mu W, Chen Z, et al (2011) Molecular characterization of the microRNA-138-Fos-like antigen 1 (FOSL1) regulatory module in squamous cell carcinoma. J Biol Chem 286: 40104–40109. 21969367 10.1074/jbc.C111.296707 PMC3220531

[32] Lerman G, Avivi C, Mardoukh C, Barzilai A, Tessone A, et al (2011) MiRNA expression in psoriatic skin: reciprocal regulation of hsa-miR-99a and IGF-1R. PLoS One 6: e20916. 21687694 10.1371/journal.pone.0020916 PMC3110257

[33] Dai Y, Zhou X (2010) Computational methods for the identification of microRNA targets. Open Access Bioinformatics 2: 29–39. 22162940 10.2147/OAB.S6902 PMC3233190

[34] Mueller AC, Sun D, Dutta A (2013) The miR-99 family regulates the DNA damage response through its target SNF2H. Oncogene 32: 1164–1172. 22525276 10.1038/onc.2012.131 PMC3407337

[35] Zheng YS, Zhang H, Zhang XJ, Feng DD, Luo XQ, et al (2012) MiR-100 regulates cell differentiation and survival by targeting RBSP3, a phosphatase-like tumor suppressor in acute myeloid leukemia. Oncogene 31: 80–92. 21643017 10.1038/onc.2011.208 PMC3253429

[36] Zhang X, Zhu T, Chen Y, Mertani HC, Lee KO, et al (2003) Human growth hormone-regulated HOXA1 is a human mammary epithelial oncogene. J Biol Chem 278: 7580–7590. 12482855 10.1074/jbc.M212050200

[37] Zhang X, Emerald BS, Mukhina S, Mohankumar KM, Kraemer A, et al (2006) HOXA1 is required for E-cadherin-dependent anchorage-independent survival of human mammary carcinoma cells. J Biol Chem 281: 6471–6481. 16373333 10.1074/jbc.M512666200

[38] Mohankumar KM, Xu XQ, Zhu T, Kannan N, Miller LD, et al (2007) HOXA1-stimulated oncogenicity is mediated by selective upregulation of components of the p44/42 MAP kinase pathway in human mammary carcinoma cells. Oncogene 26: 3998–4008. 17213808 10.1038/sj.onc.1210180

[39] Mohankumar KM, Perry JK, Kannan N, Kohno K, Gluckman PD, et al (2008) Transcriptional activation of signal transducer and activator of transcription (STAT) 3 and STAT5B partially mediate homeobox A1-stimulated oncogenic transformation of the immortalized human mammary epithelial cell. Endocrinology 149: 2219–2229. 18276758 10.1210/en.2007-1320

[40] Zha TZ, Hu BS, Yu HF, Tan YF, Zhang Y, et al (2012) Overexpression of HOXA1 correlates with poor prognosis in patients with hepatocellular carcinoma. Tumour Biol 33: 2125–2134. 22864671 10.1007/s13277-012-0472-6

[41] Ducker CE, Upson JJ, French KJ, Smith CD (2005) Two N-myristoyltransferase isozymes play unique roles in protein myristoylation, proliferation, and apoptosis. Mol Cancer Res 3: 463–476. 16123142 10.1158/1541-7786.MCR-05-0037 PMC2908404

[42] Perinpanayagam MA, Beauchamp E, Martin DD, Sim JY, Yap MC, et al (2013) Regulation of co- and post-translational myristoylation of proteins during apoptosis: interplay of N-myristoyltransferases and caspases. FASEB J 27: 811–821. 23150525 10.1096/fj.12-214924

[43] Kato U, Inadome H, Yamamoto M, Emoto K, Kobayashi T, et al (2013) Role for phospholipid flippase complex of ATP8A1 and CDC50A proteins in cell migration. J Biol Chem 288: 4922–4934. 23269685 10.1074/jbc.M112.402701 PMC3576096

[44] Wellik DM (2007) Hox patterning of the vertebrate axial skeleton. Dev Dyn 236: 2454–2463. 17685480 10.1002/dvdy.21286

[45] Iimura T, Denans N, Pourquie O (2009) Establishment of Hox vertebral identities in the embryonic spine precursors. Curr Top Dev Biol 88: 201–234. 19651306 10.1016/S0070-2153(09)88007-1 PMC3523337

[46] Alexander T, Nolte C, Krumlauf R (2009) Hox genes and segmentation of the hindbrain and axial skeleton. Annu Rev Cell Dev Biol 25: 431–456. 19575673 10.1146/annurev.cellbio.042308.113423

[47] Kachgal S, Mace KA, Boudreau NJ (2012) The dual roles of homeobox genes in vascularization and wound healing. Cell Adh Migr 6: 457–470. 23076135 10.4161/cam.22164 PMC3547888

[48] Ma L, Teruya-Feldstein J, Weinberg RA (2007) Tumour invasion and metastasis initiated by microRNA-10b in breast cancer. Nature 449: 682–688. 17898713 10.1038/nature06174

[49] Yekta S, Shih IH, Bartel DP (2004) MicroRNA-directed cleavage of HOXB8 mRNA. Science 304: 594–596. 15105502 10.1126/science.1097434

[50] Weiss FU, Marques IJ, Woltering JM, Vlecken DH, Aghdassi A, et al.. (2009) Retinoic acid receptor antagonists inhibit miR-10a expression and block metastatic behavior of pancreatic cancer. Gastroenterology 137: 2136–2145 e2131–2137. 10.1053/j.gastro.2009.08.065 19747919

[51] Ohuchida K, Mizumoto K, Lin C, Yamaguchi H, Ohtsuka T, et al (2012) MicroRNA-10a is overexpressed in human pancreatic cancer and involved in its invasiveness partially via suppression of the HOXA1 gene. Ann Surg Oncol 19: 2394–2402. 22407312 10.1245/s10434-012-2252-3

[52] Hassan NM, Hamada J, Murai T, Seino A, Takahashi Y, et al (2006) Aberrant expression of HOX genes in oral dysplasia and squamous cell carcinoma tissues. Oncol Res 16: 217–224. 17294802 10.3727/000000006783981080

[53] Bitu CC, Destro MF, Carrera M, da Silva SD, Graner E, et al (2012) HOXA1 is overexpressed in oral squamous cell carcinomas and its expression is correlated with poor prognosis. BMC Cancer 12: 146. 22498108 10.1186/1471-2407-12-146 PMC3351375

[54] Zhu T, Starling-Emerald B, Zhang X, Lee KO, Gluckman PD, et al (2005) Oncogenic transformation of human mammary epithelial cells by autocrine human growth hormone. Cancer Res 65: 317–324. 15665309

[55] Delehedde M, Cho SH, Hamm R, Brisbay S, Ananthaswamy HN, et al (2001) Impact of Bcl-2 and Ha-ras on keratinocytes in organotypic culture. J Invest Dermatol 116: 366–373. 11231309 10.1046/j.1523-1747.2001.01260.x

