# Supplementary material for: MicroRNA-99 Family Members Suppress Homeobox A1 Expression in Epithelial Cells
Source: PLoS One. 2013 Dec 3;8(12):e80625. doi: 10.1371/journal.pone.0080625 (PMC3849180; doi:10.1371/journal.pone.0080625)
Supplement: Figure S1 — siRNA-mediated down-regulation of mTOR, HOXA1, CTDSPL, NMT1, TMEM30A and SMARCA5 gene expression. 1386Ln (A) and HaCaT cells (B) were transfected with either negative control siRNA, or specific siRNAs against mTOR, HOXA1, CTDSPL, NMT1, TMEM30A or SMARCA5. The relative mRNA levels of these genes were measured by qRT-PCR. * indicates p<0.05. (PPT) [file pone.0080625.s001.ppt]

## Slide 1
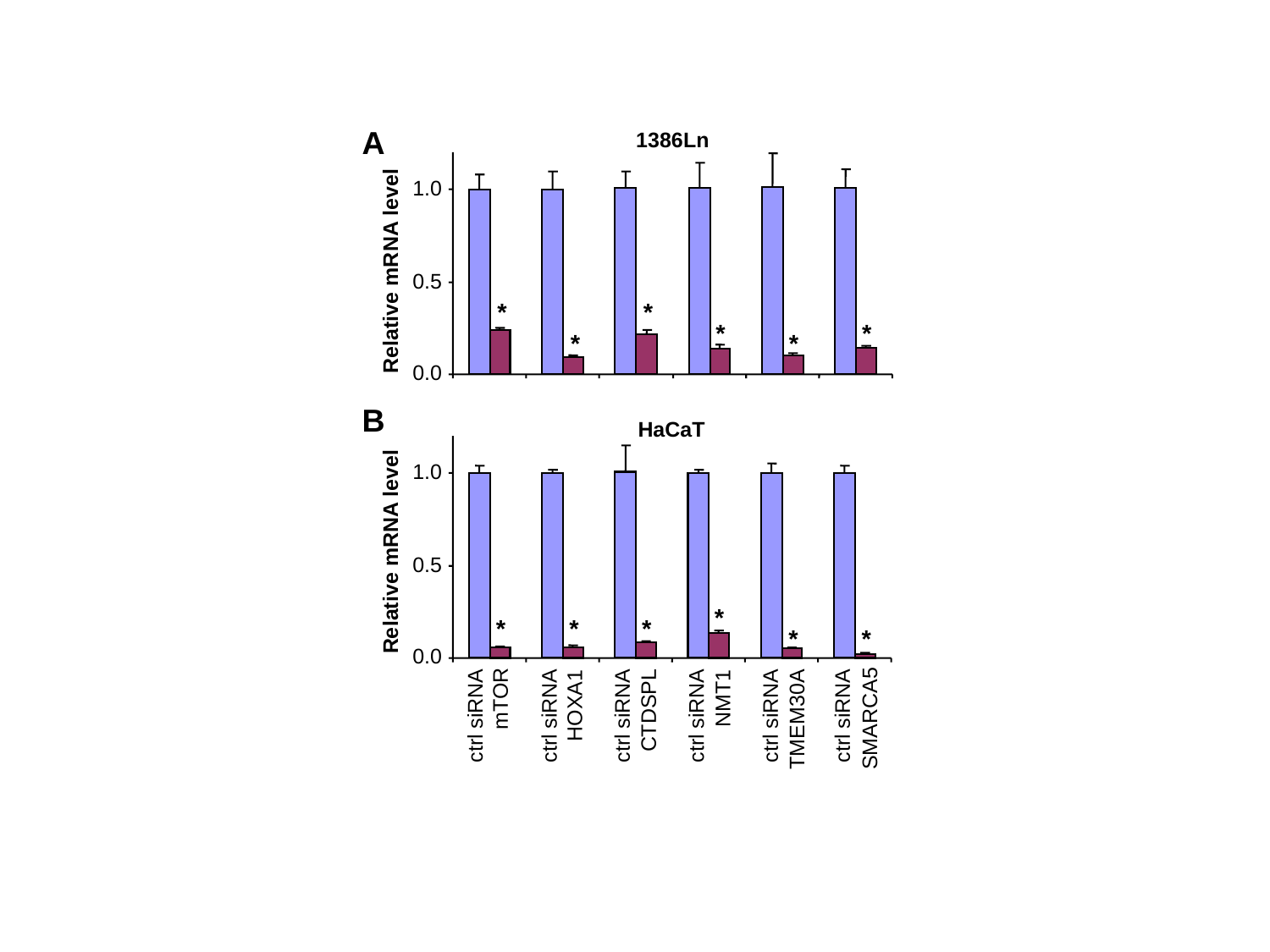

A
1386Ln
1.0
Relative mRNA level
0.5
*
*
*
*
*
*
0.0
B
HaCaT
1.0
Relative mRNA level
0.5
*
*
*
*
*
*
0.0
NMT1
mTOR
HOXA1
CTDSPL
ctrl siRNA
ctrl siRNA
ctrl siRNA
ctrl siRNA
ctrl siRNA
ctrl siRNA
SMARCA5
TMEM30A
