# Supplementary material for: MicroRNA-99 Family Members Suppress Homeobox A1 Expression in Epithelial Cells
Source: PLoS One. 2013 Dec 3;8(12):e80625. doi: 10.1371/journal.pone.0080625 (PMC3849180; doi:10.1371/journal.pone.0080625)
Supplement: Figure S2 — Predicted hsa-miR-99 family targeting sites on HOXA1 mRNAs. The predicted miR-99 family targeting sites on the mRNA sequences of (A) HOXA1 transcript variant 1 (NM_005522) and (B) HOXA1 transcript variant 2 (NM_153620). Two predicted targeting sites were identified in the transcript variant 1 of the HOXA1 gene, targeting site 1 located in the coding region and targeting site 2 located in the 3′-UTR, respectively. Only targeting site 2 was presented in the transcript variant 2 of the HOXA1 gene. The base-pairing (green: microRNA sequence; red: mRNA sequence) and the minimum free energy (mfe) for the binding of hsa-miR-100 to the targeting site 1 (C) and the targeting site 2 (D) were predicted using the RNAhybrid program [Krüger & Rehmsmeier: RNAhybrid: microRNA target prediction easy, fast and flexible. Nucleic Acids Res. 2006 Jul 1;34(Web Server issue):W451–4]. (PPT) [file pone.0080625.s002.ppt]

## Slide 1
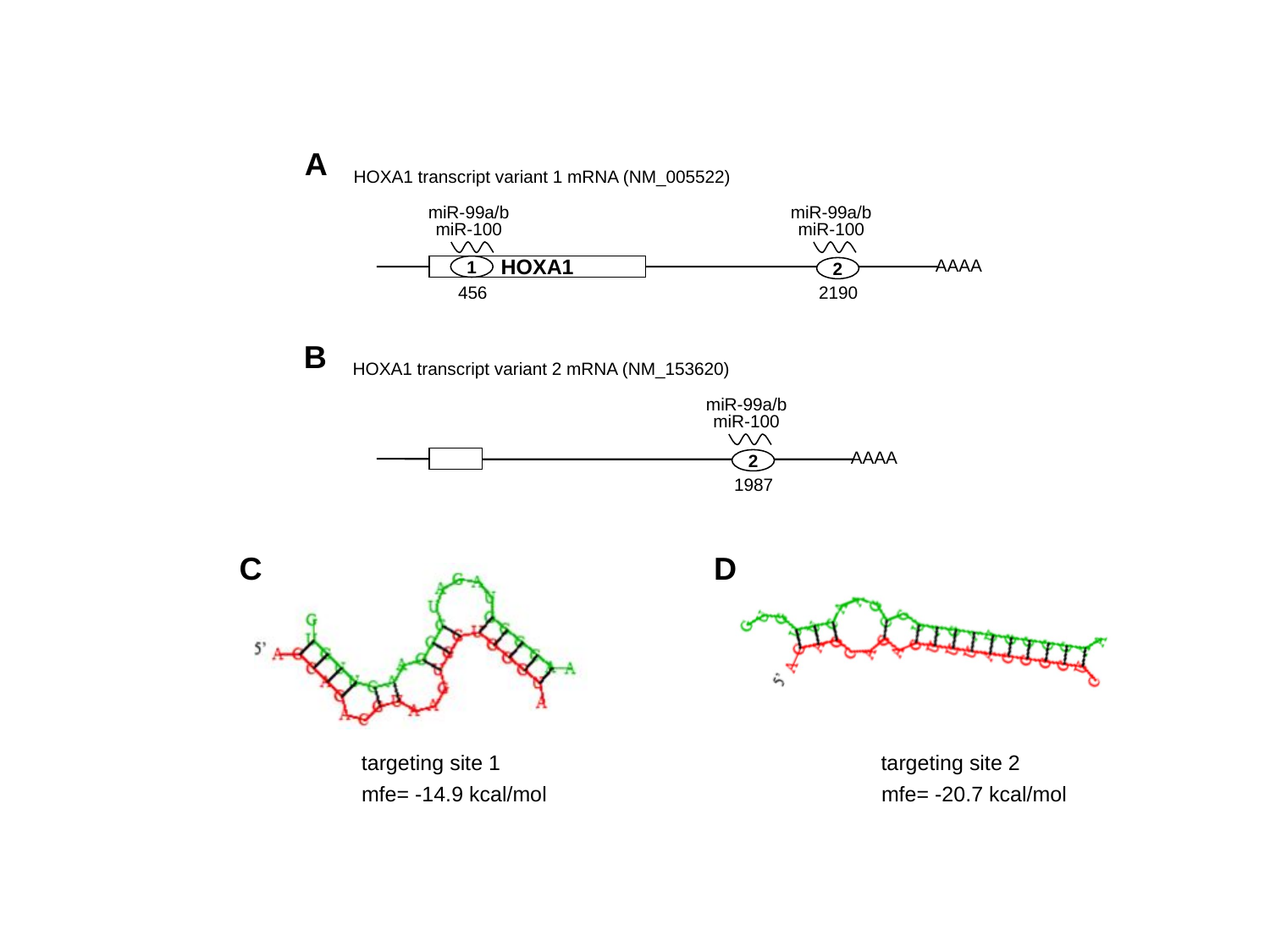

A
HOXA1 transcript variant 1 mRNA (NM_005522)
miR-99a/b
miR-100
miR-99a/b
miR-100
AAAA
HOXA1
1
2
2190
456
B
HOXA1 transcript variant 2 mRNA (NM_153620)
miR-99a/b
miR-100
AAAA
2
1987
C
D
targeting site 1
targeting site 2
mfe= -14.9 kcal/mol
mfe= -20.7 kcal/mol
