# Supplementary material for: MicroRNA-99 Family Members Suppress Homeobox A1 Expression in Epithelial Cells
Source: PLoS One. 2013 Dec 3;8(12):e80625. doi: 10.1371/journal.pone.0080625 (PMC3849180; doi:10.1371/journal.pone.0080625)
Supplement: Figure S3 — Predicted hsa-miR-99 family targeting sites on HOXA3 mRNA. (A) Two predicted targeting sites were predicted in the HOXA3 mRNA (NM_030661), located in the 5′-UTR and coding region, respectively. The base-pairing (green: microRNA sequence; red: mRNA sequence) and the minimum free energy (mfe) for the binding of hsa-miR-100 to the targeting site 1 (B) and the targeting site 2 (C) were predicted using the RNAhybrid program [Krüger & Rehmsmeier: RNAhybrid: microRNA target prediction easy, fast and flexible. Nucleic Acids Res. 2006 Jul 1;34(Web Server issue):W451–4]. (PPT) [file pone.0080625.s003.ppt]

## Slide 1
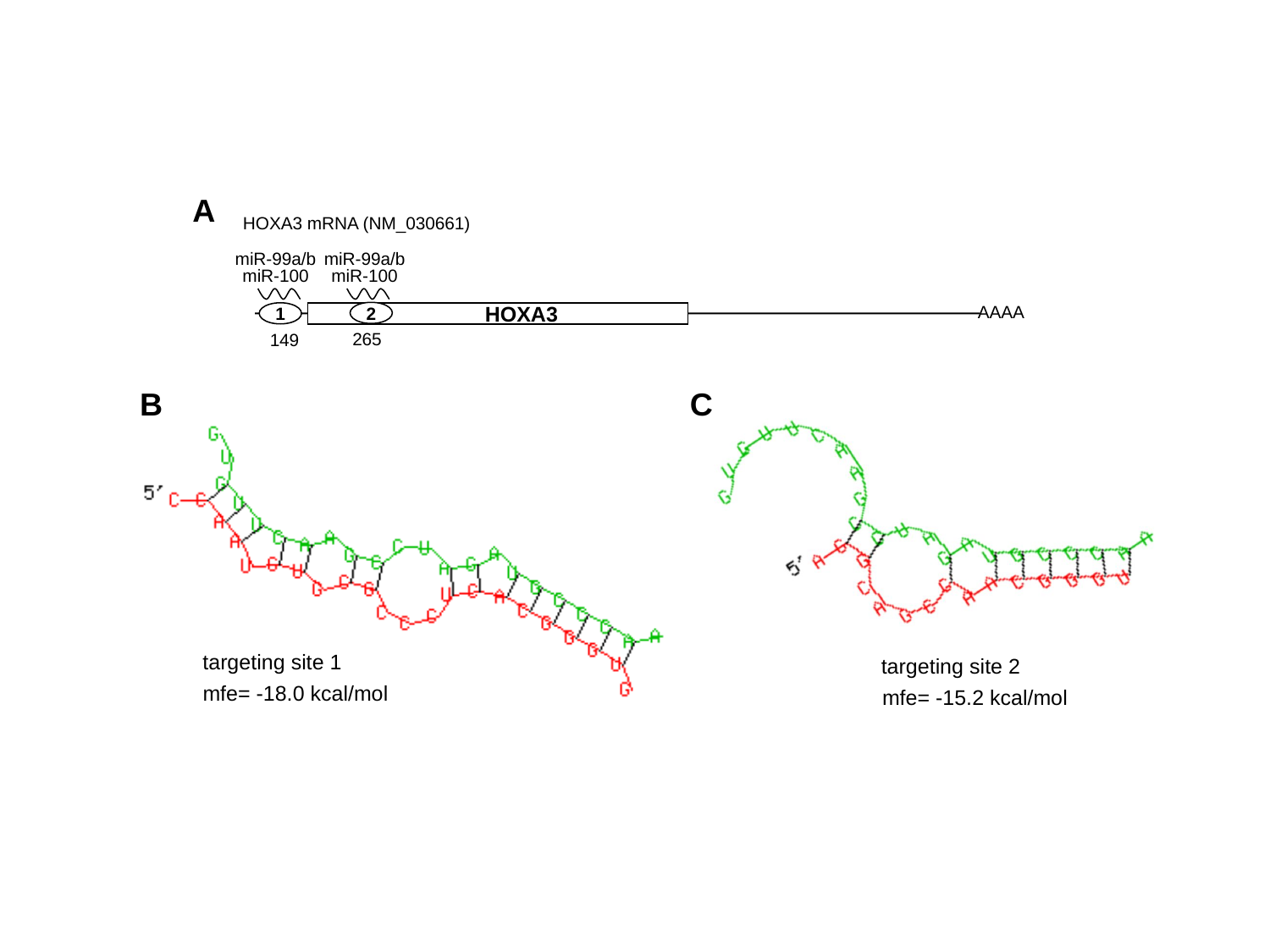

A
HOXA3 mRNA (NM_030661)
miR-99a/b
miR-100
miR-99a/b
miR-100
AAAA
2
1
 HOXA3
265
149
B
C
targeting site 1
mfe= -18.0 kcal/mol
targeting site 2
mfe= -15.2 kcal/mol
