# Supplementary figures and images for: MicroRNA-99 Family Members Suppress Homeobox A1 Expression in Epithelial Cells
Source: PLoS One. 2013 Dec 3;8(12):e80625. doi: 10.1371/journal.pone.0080625 (PMC3849180; doi:10.1371/journal.pone.0080625)

## Slide 1
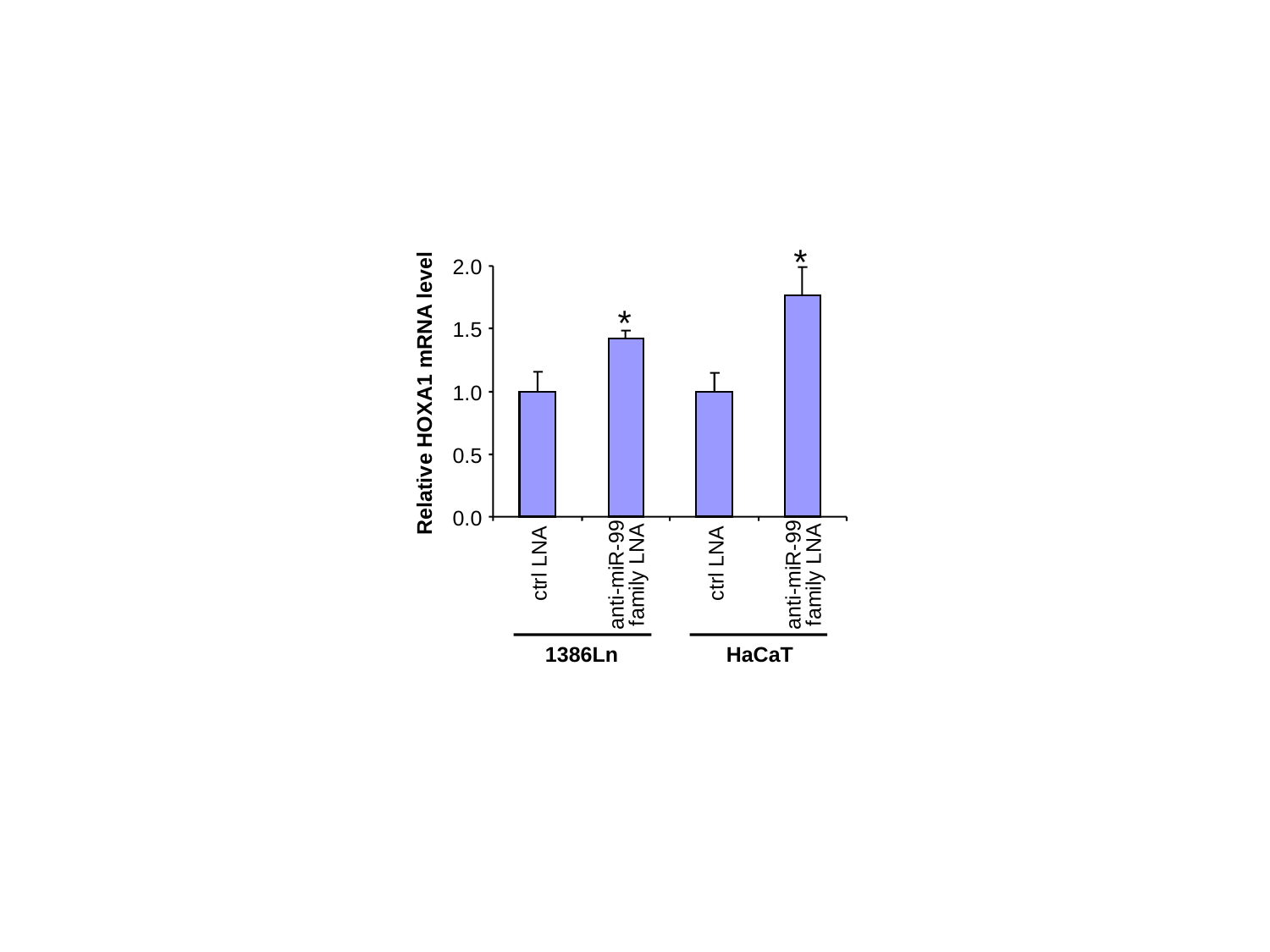

*
2.0
1.5
1.0
0.5
0.0
*
Relative HOXA1 mRNA level
ctrl LNA
ctrl LNA
anti-miR-99
family LNA
anti-miR-99
family LNA
1386Ln
HaCaT

Supplement: Figure S4 — The effect of miR-99 family LNA inhibitor on HOXA1 expression. 1386Ln and HaCaT cells were treated with LNA inhibitor for miR-99 family, or negative control LNA. The expression of HOXA1 gene was examined by qRT-PCR. *: p<0.05. (PPT) [file pone.0080625.s004.ppt]
